# Δ12 fatty acid desaturase gene from *Geotrichum candidum* in cheese: molecular cloning and functional characterization

**DOI:** 10.1002/2211-5463.12553

**Published:** 2018-12-06

**Authors:** Xue Luo, Haisu Shi, Rina Wu, Junrui Wu, Yuzhen Pi, Yan Zheng, Xiqing Yue

**Affiliations:** ^1^ College of Food Science Shenyang Agricultural University China

**Keywords:** cheese, delta 12 fatty acid desaturase, *Geotrichum candidum*

## Abstract

Soft cheese with white rind lacks essential fatty acids (EFAs), and as a result its long‐term consumption may lead to various kinds of cardiovascular and cerebrovascular diseases, such as hyperlipidemia, hypertension, and atherosclerosis. *Geotrichum candidum* is a dimorphic yeast that plays an important role in the ripening of mold cheese. A gene coding for Δ12 fatty acid desaturase, a critical bifunctional enzyme desaturating oleic acid (OA) and linoleic acid (LA) to produce LA and α‐linolenic acid (ALA), respectively, was isolated from *G. candidum*, and then cloned and heterologously expressed in *Saccharomyces cerevisiae*. This gene, named *GcFADS12*, had an open reading frame of 1257 bp and codes for a protein of 419 amino acids with a predicted molecular mass of 47.5 kDa. Characterization showed that GcFADS12 had the ability to convert OA to LA and LA to ALA, and the conversion rates for OA and LA were 20.40 ± 0.66% and 6.40 ± 0.57%, respectively. We also found that the protein product of *GcFADS12* catalyzes the conversion of the intermediate product (LA) to ALA by addition of OA as the sole substrate. The catalytic activity of GcFADS12 on OA and LA was unaffected by fatty acid concentrations. Kinetic analysis revealed that GcFADS12 had stronger affinity for the OA than for the LA substrate. This study offers a solid basis for improving the production of EFAs by *G. candidum* in cheese.

**Abbreviations**
ALA: α‐linolenic acid (18:3^Δ9,12,15^)
EFA: essential fatty acid
FADS6: Δ6 fatty acid desaturase
FADS12: Δ12 fatty acid desaturase
FADS15: Δ15 fatty acid desaturase
*G. candidum*: *Geotrichum candidum*
LA: linoleic acid (18:2^Δ9,12^)
OA: oleic acid (18:1^Δ9^)
PUFA: polyunsaturated fatty acid

The industry of making mold cheese, a kind of cheese fermented by mold, is in its initial stage in China 1. Mold cheese contains rich nutrients such as protein, calcium, fatty acids and vitamins, among which fatty acids account for 24–28%. Fatty acids in mold cheese play an important role in the formation of cheese flavors and are especially indispensable for the human diet 2. However, cheese lacks polyunsaturated fatty acids (PUFAs), especially essential fatty acids (EFAs) including linoleic acid (LA, 18:2^Δ9,12^) and α‐linolenic acid (ALA, 18:3^Δ9,12,15^), and this problem has not been solved so far. This has been the main reason why the proportion of saturated and unsaturated fatty acids in cheese is seriously unbalanced. Therefore, the long‐term consumption of cheese containing a large amount of saturated fatty acids and lacking EFAs may lead to various kinds of cardiovascular and cerebrovascular diseases such as hyperlipidemia, hypertension and atherosclerosis.

*Geotrichum candidum*, a dimorphic yeast commonly used as a starter for cheese making, is a dominant strain that plays an important role in the ripening of mold cheese and in forming the unique appearance, special aroma and taste of soft cheese with white rind 3, 4, 5, 6. *G. candidum* is safe for food use; its safety has been studied since the 1960s and its fermented products have a high level of safety for consumers 7. *G. candidum* has been used widely to produce lipase 8, 9, 10, but it has not been applied in EFA biosynthesis in the mold cheese‐making industry thus far.

In EFA biosynthesis, Δ12 fatty acid desaturase (FADS12), Δ15 fatty acid desaturase (FADS15) and Δ6 fatty acid desaturase (FADS6) play a key role in regulating the level of EFAs. In a previous study, the molecular mechanism, substrate specificity and catalytic activity for FADS6 were analyzed 11, 12 and applied to PUFA synthesis 13, 14. FADS12 is a key bifunctional membrane‐bound desaturase that converts oleic acid (OA, 18:1^Δ9^) to linoleic acid (LA, 18:2^Δ9,12^) and LA to α‐linolenic acid (ALA, 18:3^Δ9,12,15^) by introducing a double bond between the carbons 12 and 13 from the carboxyl end of the substrate in the biosynthesis of EFAs 15, 16, 17. The genome of *G. candidum* has been sequenced and submitted to the GenBank database (LOCUS: CCBN010000001) by Casaregola, and we found from its genome sequence that there was a sequence encoding Δ12 fatty acid desaturase of *G. candidum* (*GcFADS12*). However, its function has been not analyzed. The level of OA, as a substrate of FADS12, accounts for about one‐quarter of the total fatty acid in mold cheese. Many studies have reported that the *FADS12* gene from various species is overexpressed for LA production, and the relative LA level was increased up to fivefold under flask culture conditions in *Rhodosporidium toruloides* 16, 18, 19. It was inferred that the catalytic properties of FADS12 directly determine the EFA level in mold cheese. Thus, it is necessary to clone the *GcFADS12* gene and characterize the function of GcFADS12.

In the present study, the relative transcript level of the *GcFADS12* gene of *G. candidum* was determined. Furthermore, the *GcFADS12* gene was cloned, and the function of the gene product characterized following expression in *Saccharomyces cerevisiae*. In addition, multiple sequence alignment of *FADS12s* and a phylogenetic analysis were performed. Finally, the effect of substrate concentration on the conversion rate and kinetics of GcFADS12 was analyzed to assess its affinity for both substrates.

## Materials and methods

### Strains and plasmids

*Geotrichum candidum* was isolated from mold cheese and preserved in our Animal‐derived Foods Processing and Utilization lab. Plasmid pYES2/NT C (Invitrogen, Shanghai, China) with 6 × His tag was used for expression of the *GcFADS12* gene and detection of *GcFADS12*.

### Media and cultural conditions

LB agar plates for plasmid construction in *Escherichia coli* Top 10 and SC‐U synthetic minimal medium for gene expression in *S. cerevisiae* were used as described previously 12. *G. candidum* was grown on Yeast Peptone Dextrose medium (containing 10 g·L^−1^ yeast extract, 10 g·L^−1^ peptone, 10 g·L^−1^ glucose and 20 g·L^−1^ agar) at 28 °C.

### RNA isolation and RT‐qPCR analysis

Approximately 0.5 g (dry weight) of *G. candidum* cells was ground to a fine powder with a precooled mortar and pestle using liquid nitrogen. Total RNA was isolated using the RNAprep Pure Plant Kit, and reverse‐transcribed with QuantScript RT Kit as described previously 13. RT‐qPCR was performed as described previously 14, and the transcript levels were calculated using the $2^{-\Delta \Delta C_{t}}$ method.

### Primer design, PCR amplification and sequence analysis for the *GcFADS12* gene

To identify genes encoding GcFADS12, a PCR‐based cloning strategy was adopted. According to the available sequence information of FADS12 from *Galactomyces candidum* WGS (protein id: CDO51572.1; GenBank accession no. for its genome: CCBN010000001), two highly degenerate primers (shown in Table S1) were designed to target sequences corresponding to the first and the third His‐rich motif in GcFADS12. Two primers were designed to clone the upstream sequence from ‘HGKHHK’ and the downstream sequence from ‘HDIIETHVLHH’ (shown in Table S1). After the full‐length cDNA of *GcFADS12* was amplified, it was ligated into pMD19‐T simple vector and sequenced.

### Yeast transformation, heterologous expression of the *GcFADS12* gene in *S. cerevisiae* and western blot analysis of GcFADS12

Plasmid pYES2/NT C‐*GcFADS12* was transformed into *S. cerevisiae* and induced. After induction, cultures were supplemented with 0.25 mm *cis*‐OA or 0.25 mm *cis*‐LA and 1% Tergitol Nonidet P‐40 for the solubilization of fatty acids. For substrate concentration experiments, 0.125, 0.25 or 0.5 mm *cis*‐OA or *cis*‐LA was added in the induced medium. For western blotting analysis, THE^™^ His tag antibody (Invitrogen) and horseradish peroxidase‐conjugated goat anti‐mouse IgG (H&L) (Bio‐Rad, Shanghai, China) were used for *GcFADS12* detection. The protocol of induction, transformation and western blotting used was the same as described previously 11.

### Phylogenetic analysis and topology prediction

Multiple sequence alignment of *FADS12s* from various species was performed and a phylogenetic tree was constructed using dnaman software (Lynnon, Suite D, San Ramon, CA, USA) and visualized with treeview. A predicted topology model for *GcFADS12* was performed with TMHMM.

### Lipid extraction and fatty acid analysis

Lipids from an equivalent weight of cells were extracted and methyl esterified, and fatty acid content was analyzed by gas chromatography as described previously 14.

## Results

### Relative transcript level of the *GcFADS12* gene in *Geotrichum candidum*

**Figure 1:**
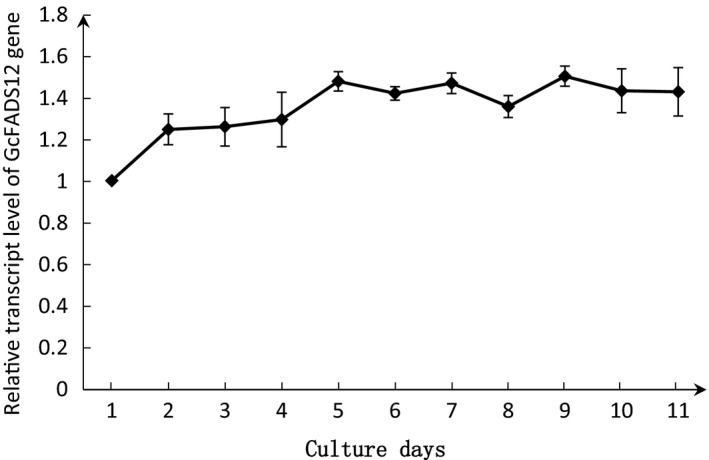
Relative transcript level of *GcFADS12* gene in *Geotrichum candidum* for 11‐day cultures. All values are the means of three independent experiments and error bars represent standard deviation. Relative transcript level of *GcFADS12* gene after growth of *G. candidum* for the first day was defined as 1.

To analyze whether the relative transcript level of the *GcFADS12* gene is stable during various fermentations of *G. candidum*, RT‐qPCR was performed. The results revealed that the transcript level of the *GcFADS12* gene in *G. candidum* had no significant difference among various fermentation periods with a slight increase that ranged from 1.2 to 1.5 (Fig. 1), suggesting that *GcFADS12* relative transcript level was stable in culture of *G. candidum*.

### Cloning of the *GcFADS12* gene

To clone the *GcFADS12* gene, a 600‐bp DNA fragment was amplified from our previously screened *G. candidum*, using degenerate primers derived from highly conserved histidine sequences of Δ12 fatty acid desaturase. Cloning of the full‐length *GcFADS12* gene was achieved by PCR amplification from the flanking regions of *G. candidum*. The gene was named *GcFADS12* and had an open reading frame of 1257 bp coding for 419 amino acids with an average molecular mass of 47.5 kDa. The gene was confirmed by sequencing and submitted to the GenBank database (LOCUS: MH198047).

### Multiple sequence alignment and phylogenetic analysis of *FADS12s*

**Figure 2:**
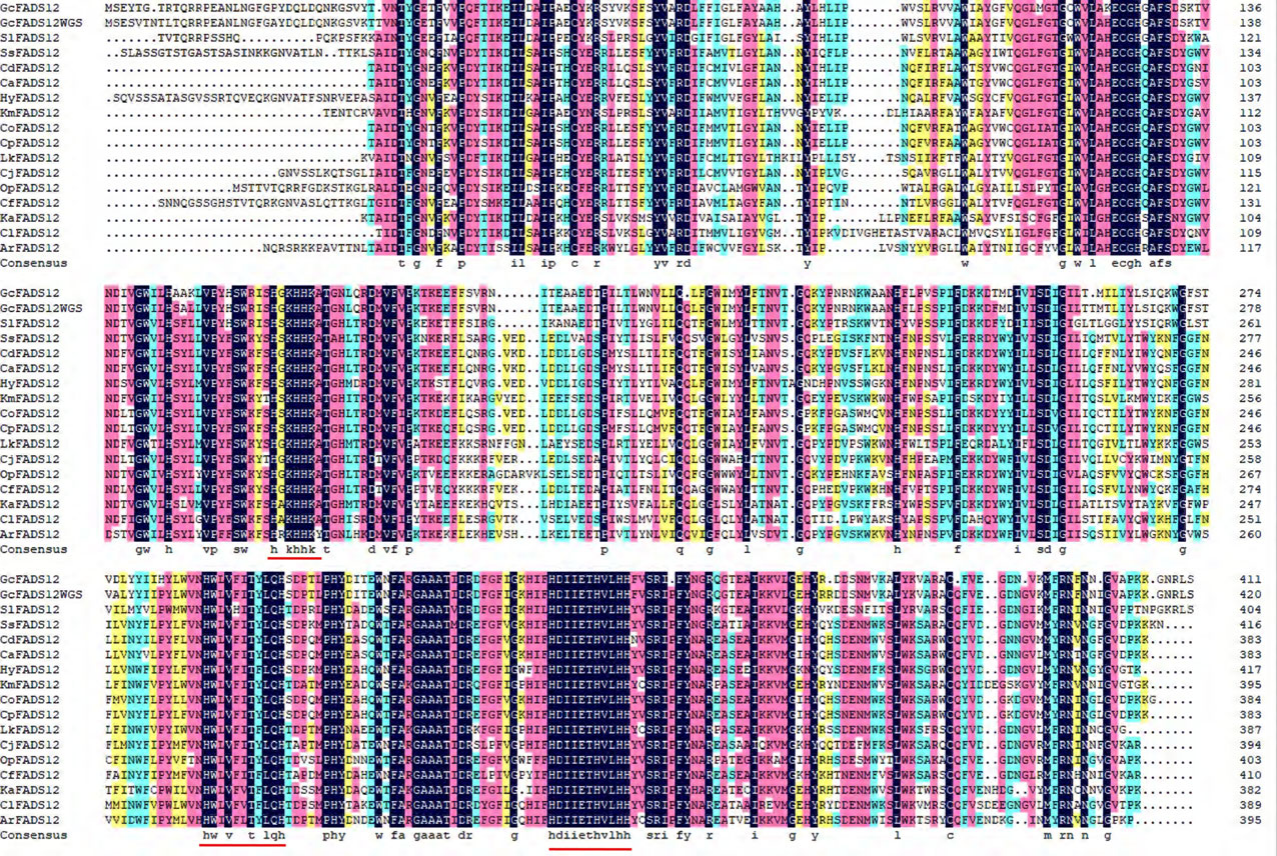
Multiple sequence alignment of deduced amino acids for *GcFADS12* with *FADS12s* from other species using dnaman software. The three conserved histidine‐rich motifs are underlined in red. *Galactomyces candidum* WGS,* GcFADS12* WGS;* Sugiyamaella lignohabitans*,* SlFADS12*;* Scheffersomyces stipitis*,* SsFADS12*;* Candida dubliniensis*,* CdFADS12*;* Candida albicans*,* CaFADS12*;* Hyphopichia burtonii*,* HyFADS12*;* Kluyveromyces marxianus*,* KmFADS12*;* Candida orthopsilosis*,* CoFADS12*;* Candida parapsilosis*,* CpFADS12*;* Lachancea kluyveri*,* LkFADS12*;* Cyberlindnera jadinii*,* CjFADS12*;* Ogataea parapolymorpha*,* OpFADS12*;* Cyberlindnera fabianii*,* CfFADS12*;* K. marxianus*,* KaFADS12*;* Scheffersomyces stipites*,* SsFADS12*;* Clavispora lusitaniae*,* ClFADS12*;* Ascoidea rubescens*,* ArFADS12*.

The amino acid sequence of *GcFADS12* showed a significant similarity (66.44%) to Δ12 fatty acid desaturase of several species in a multiple sequence alignment (Fig. 2). In addition, the *GcFADS12* sequence had 94.30% identity with *FADS12* from *G. candidum* WGS.

**Figure 3:**
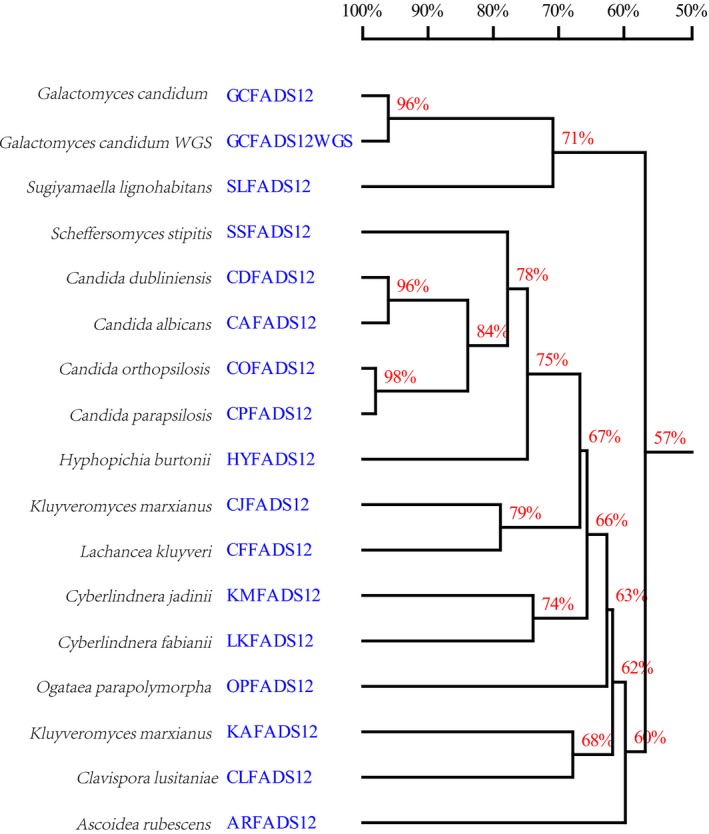
Phylogenetic analysis of the putative *GcFADS12* sequence and *FADS12* sequences from other species. The construction of the unrooted phylogenetic tree was performed using dnaman software. The branch length and the identity of each *FADS12* sequence were generated by the phylogenetic tree and homology tree program, respectively.

A phylogenetic tree was constructed for *GcFADS12* and other *FADS12s* from various species. Phylogenetic analysis of the putative *GcFADS12* gene showed that it had a close relationship with the *FADS12* gene sequence in *G. candidum* WGS. *SlFADS12* and both *GcFADS12s* clustered in a major clade, *FADS12s* from *Kluyveromyces marxianus*,* Clavispora lusitaniae and Ascoidea rubescens* formed a separate cluster at the bottom of the tree, and other *FADS12s* were in another group (Fig. 3).

### Characterization of *GcFADS12* function

**Figure 4:**
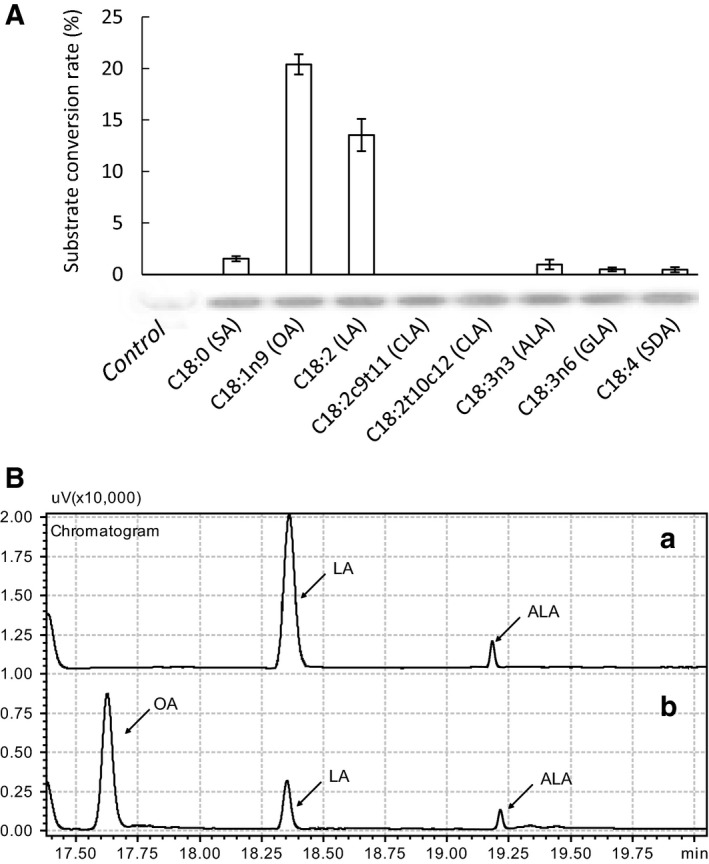
(A) Substrate conversion rate of GcFADS12 protein expressed in *Saccharomyces cerevisiae*, determined by adding 0.25 mm SA, 0.25 mm OA, 0.25 mm LA, 0.25 mm CLA, 0.25 mm ALA, 0.25 mm GLA or 0.25 mm ALA after induction with 2% galactose. Substrate conversion rate = 100 × ([product]/[product + substrate]). Bars show the means of three independent samples (three transformants in SC‐U plates) and error bars represent standard deviation. Western blot of each sample is shown below the corresponding bar. (B) Gas chromatogram of fatty acids of GcFADS12 protein expressed in *S. cerevisiae* with addition of 0.25 mm LA (a) and 0.25 mm OA (b).

The expression level of the *GcFADS12* gene in *S. cerevisiae* was determined by SDS/PAGE and western blot. The GcFADS12 band was generally not visible in SDS/PAGE as the expression of the membrane enzyme was low in *S. cerevisiae*. But western blot analysis showed that there was a specific protein band with a molecular mass of 50 kDa, which was in accordance with the theoretical molecular mass; and there was no significant difference among various samples (Fig. 4A).

The function of GcFADS12 was characterized by adding various fatty acid substrates. The results of gas chromatography showed that it had the function of FADS12, but its ability to transform OA and LA substrates was weak (Fig. 4A,B). These findings revealed that *GcFADS12* encoded a fatty acid desaturase possessing FADS12 activity, which could successfully convert exogenous OA to LA and LA to ALA. The conversion rate from OA to LA and LA to ALA was 20.40 ± 0.66% and 6.40 ± 0.57%, respectively (Fig. 4A). In addition, GcFADS12 also continued to transform LA (the product of OA) to ALA by addition of OA as the substrate, and the conversion rate from LA to ALA was 9.80 ± 0.25% (Table 1).

**Table 1:** Fatty acid concentration (mm) of yeast transformants harboring the control plasmid (pYES2/NT C) and the recombinant plasmid (pYES2‐*GcFADS12*) by adding 0.125/0.250/0.500 mm *cis*‐OA or 0.125/0.250/0.500 mm *cis*‐LA as fatty acid substrates. Data are means ± standard deviation calculated from three independent samples. ND, not detected. OA conversion rate = 100 × ([product of LA + ALA]/substrate addition). LA conversion rate = 100 × ([product of ALA]/product of LA + ALA)

Substrate and concentration (mm)		Remaining substrate (mm)	Production of substrate (mm)		OA conversion rate (%)	LA conversion rate (%)
			LA	ALA		
pYES2/NT C transformants						
OA	0.125	0.124 ± 0.009 (OA)	ND	ND	ND	–
	0.250	0.248 ± 0.005 (OA)	ND	ND	ND	–
	0.500	0.494 ± 0.007 (OA)	ND	ND	ND	–
LA	0.125	0.123 ± 0.003 (LA)	–	ND	–	ND
	0.250	0.249 ± 0.008 (LA)	–	ND	–	ND
	0.500	0.497 ± 0.015 (LA)	–	ND	–	ND
pYES2‐*GcFADS12* transformants						
OA	0.125	0.090 ± 0.005 (OA)	0.028 ± 0.008	0.003 ± 0.000	25.04 ± 0.45	10.54 ± 0.08
	0.250	0.198 ± 0.007 (OA)	0.046 ± 0.009	0.005 ± 0.000	20.40 ± 0.66	9.80 ± 0.25
	0.500	0.390 ± 0.014 (OA)	0.080 ± 0.019	0.009 ± 0.001	17.72 ± 1.02	9.71 ± 0.54
LA	0.125	0.109 ± 0.006 (LA)	–	0.009 ± 0.001	–	7.12 ± 0.36
	0.250	0.233 ± 0.010 (LA)	–	0.016 ± 0.002	–	6.40 ± 0.57
	0.500	0.450 ± 0.019 (LA)	–	0.029 ± 0.004	–	5.80 ± 0.84

### The effect of substrate concentration on the conversion rate for GcFADS12

To characterize the effect of substrate concentration on the conversion rate of *GcFADS12*,* S. cerevisiae* transformants harboring pYES2/NT C‐*GcFADS12* plasmid and the control (harboring pYES2/NT C) were incubated with 0.125/0.250/0.500 mm OA or 0.125/0.250/0.500 mm LA as fatty acid substrates for 12 h at 28 °C, and the resulting fatty acid composition is listed in Table 1. The results revealed that substrate concentration had little effect on the conversion rate in yeast expressing *GcFADS12*, whereas there was significantly augmented OA conversion from 25.04 ± 0.45% to 17.72 ± 1.02% and LA conversion from 10.54 ± 0.08% to 9.71 ± 0.54% by OA addition. LA conversion ranged from 7.12 ± 0.36% to 5.80 ± 0.84% by LA addition (Table 1).

### Kinetics of GcFADS12

The affinity of GcFADS12 was next assessed by evaluating its reaction rate in the presence of two fatty acid concentrations of OA or LA, where the values of maximal reaction velocity (*V*_m_) and *K*_m_ were calculated using the Michaelis–Menten equation (Table 2). The kinetics results showed that the *K*_m_ value for OA as substrate was lower than that for LA as substrate for the pYES2‐*GcFADS12* transformant, whereas *V*_m_ for OA as substrate was more than double that for LA as substrate (Table 2). In addition, the *K*_m_ value for LA as substrate, which was the intermediate product of OA of GcFADS12, was markedly decreased, compared to that for LA as the substrate; but it corresponded to that for OA as the substrate. The *V*_m_ for LA as substrate (the intermediate product of OA) was much lower than that for OA as the substrate, as there was a low quantity of the intermediate product of LA (Table 2). Together, these results suggest that GcFADS12 had stronger affinity for OA than for LA as substrate.

**Table 2:** Kinetic analysis of GcFADS12. *V*_m_ and *K*_m_ were calculated by the Michaelis–Menten equation. IP, intermediate product

Fatty acid concentration (mm)	*V* (μm·min^−1^)			*V*_m_ (μm·min^−1^)			*K*_m_		
	OA	LA (IP)	LA	OA	LA (IP)	LA	OA	LA (IP)	LA
0.250	0.071	0.007	0.016	0.47	0.04	0.21	1403.0	1285.7	2166.7
0.500	0.123	0.012	0.040						

## Discussion

**Figure 5:**
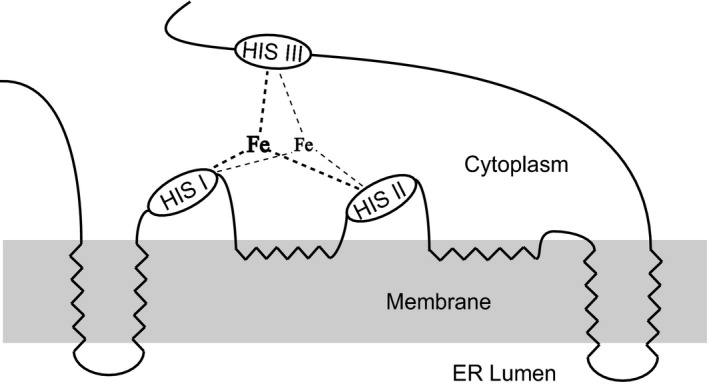
Skeleton map of the amino acid sequences of GcFADS12. Polygonal lines show each transmembrane domain, and ovals indicate three histidine‐rich conserved motifs.

In this study, *FADS12* cDNA from *G. candidum* (*GcFADS12*) was cloned and its functional characterization was reported. Based on the multiple sequence alignment of the other FADS12 enzymes, the functional domains of GcFADS12 were highly conserved among various species (Fig. 2). GcFADS12 possessed typical characteristics of other FADS12 family members, including three histidine‐rich conserved motifs (HXXHHK, HWXVXXTXLQH and HLVHH) and six transmembrane domains (Fig. 5), and these observations were in agreement with our previous reports on Δ6 fatty acid desaturase 12, 16. The amino acid sequence of GcFADS12 was highly similar to the one that S. Casaregola submitted to the GenBank (protein id: CDO51572.1), which was in agreement with the observation from our phylogenetic analysis of *FADS12* (Fig. 3).

The bifunctional FADS12 enzyme has been widely characterized as being responsible for converting the OA and LA fatty acid substrates to LA and ALA, respectively, in many species 17, 20, 21: functional analysis results showed that FADS12 from *Rhodosporidium kratochvilovae* converted 11.4% of OA to LA, and 19.1% of LA to ALA; Wei *et al*. 20 reported that FADS12 from *Rhizopus arrhizus* had a high conversion rate of 16.0% for OA when it was expressed in yeast; and the conversion rate of OA by FADS12 from *Mortierella alpina* 1S‐4 was approximately 24.1%, and that of LA was 36.8%. FADS12 isolated from *G. candidum* in cheese in this study was also bifunctional for both OA and LA, and we found from our functional analysis that GcFADS12 should continually catalyze the product (LA) of OA to ALA. This plays an important role in enhancing and especially balancing the level of essential fatty acids (LA and ALA) from *G. candidum* in cheese.

We found that the conversion rates of OA and LA for GcFADS12 showed a downward trend as the substrate concentration (as shown in Table 1). However, as the extent of the decline became smaller, it conformed to the curve of the Michaelis–Menten equation. The kinetics of GcFADS12 showed that it had a strong affinity and conversion rate for OA, which was consistent with the results of GcFADS12's functional characterization.

In this study, we newly isolated and identified a gene coding for Δ12 fatty acid desaturase from *G. candidum*, characterized its function with OA and LA, and assessed its affinity for both substrates. Our study presents a step forward to producing EFAs with *G. candidum* in cheese.

## Conflict of interest

The authors declare no conflict of interest.

## Author contributions

HS designed and carried out this work, and drafted the manuscript. XL analyzed the data and helped to draft the manuscript. RW and JW supervised the research and helped to draft the manuscript. YP, YZ and XY conceived the study and revised the manuscript. All authors read and approved the final manuscript.

## Supporting information


**Table S1.** Primers used in this study.
