# Supplementary material for: Δ12 fatty acid desaturase gene from Geotrichum candidum in cheese: molecular cloning and functional characterization
Source: FEBS Open Bio. 2018 Dec 6;9(1):18–25. doi: 10.1002/2211-5463.12553 (PMC6325598; doi:10.1002/2211-5463.12553)
Supplement: Supplementary file 1 — Table S1. Primers used in this study. [file FEB4-9-18-s001.doc]

**Supplemental Table S1** Primers used in this study

| Primer name | Oligonucleotide sequence (5'-3') | Function |
| --- | --- | --- |
| F*Gc-1* | TC(T/C/A/G)CA(T/C)GG(T/C/A/G)AA(A/G)CA(T/C)CA(T/C)AA(A/G) | Amplification for the first and the third His-rich motif of *GcFADS12* |
| R*Gc-1* | GT(T/C/A/G)GT(T/C/A/G)GA(A/G)CA(T/C)GT(T/C/A/G)AG(T/C)CT(T/C/A/G) |
| F*Gc-2* | CTTGTGGTGCTTACCGTG | Amplification for the upstream sequence from “HGKHHK” and the downstream sequence from “HDIIETHVLHH” |
| R*Gc-2* | GAGACTCACGTACTGCACCAC |
| T7 | TAATACGACTCACTATAGGG | Target genes insert detection for yeast expression construction |
| pYES2.R | TCGGTTAGAGCGGATGTG |
| RT-*Gc*D12 | GCGAGTGCCAGTGTACTCAGACAT | qPCR for *GcFADS12* transcript level measurement |
| RT-*Gc*D12 | GGCGTCGAGAATTTCCTTGA |
| 18SRTF | GGCAAGTCTGGTGCCAGCAGC | The control gene for qPCR |
| 18SRTR | TTGGCAAATGCTTTCGC |
